# Molecular characterization of *Escherichia coli* recovered from traditional milk products in Kashan, Iran

**DOI:** 10.14202/vetworld.2017.1264-1268

**Published:** 2017-10-24

**Authors:** Farhad Sharafati Chaleshtori, Navid Mazroii Arani, Esmat Aghadavod, Afshin Naseri, Reza Sharafati Chaleshtori

**Affiliations:** 1 Department of Microbiology, Medical Plants Research Center, Basic Health Sciences Institute, Shahrekord University of Medical Sciences, Shahrekord, Iran; 2 Department of Microbiology Food and Hygiene Control Laboratory, Deputy of Food and Drug, Kashan University of Medical Sciences, Kashan, Iran; 3 Department of Biochemistry, Research Center for Biochemistry and Nutrition in Metabolic Diseases, Kashan University of Medical Sciences, Kashan, Iran; 4 Department of Nutrition, Research Center for Biochemistry and Nutrition in Metabolic Diseases, Kashan University of Medical Sciences, Kashan, Iran

**Keywords:** *Escherichia coli*, milk products, molecular characterization

## Abstract

**Aim:**

Shiga toxigenic *Escherichia coli* (STEC) strains as emerging groups of foodborne pathogens are responsible for most foodborne illnesses. The aim of this study was to determine the antibiotic resistance pattern in STEC isolated from traditional milk products and their molecular characterization.

**Materials and Methods:**

A total of 116 samples were randomly purchased from local markets in Kashan, Iran, and evaluated for the occurrence of STEC by culturing and molecular methods. The antibiotic resistance of obtained isolates was determined by Kirby Bauer method. Furthermore, isolates were assayed for the presence of Shiga toxins (*stx1* and *stx2*) and intimin gene (*eae*).

**Results:**

The incidence of *E. coli* in 60 ice cream, 30 yoghurt, and 26 cheese samples was 8.33%, 10%, and 11.54%, respectively. The findings showed that 11 out of 11 (100%) *E. coli* had both *stx1* and *stx2* while *eae* gene was not found in *E. coli* isolated of traditional milk products. For *E. coli* strains carrying *stx1* and *stx2*, highest antibiotic sensitive levels were related to trimethoprim/sulfamethoxazole, norfloxacin, chloramphenicol, and ciprofloxacin, respectively.

**Conclusion:**

The results showed relationship between the presence of virulence factors and antimicrobial resistance. These results can be used for further studies on STEC as an emerging foodborne pathogen.

## Introduction

Raw milk products such as traditional cheese, ice cream, and yoghurt can be a main source of potentially harmful bacteria to human, such as *Escherichia coli*. Foodborne disease outbreaks have been reported by consumption of raw milk and raw milk products in developing and developed countries [1]. In Iran, traditional milk products are consumed, especially in some rural and urban areas.

*E. coli* is one of the most important pathogenic bacteria, which is a normal inhabitant of large intestine in human and warm-blooded animals [2]. Therefore, *E. coli* can be transmitted to raw milk and milk products by fecal contamination during milking process along with poor hygienic practices [3,4].

In recent years, Shiga toxigenic *E. coli* (STEC) strains as emerging groups of foodborne pathogens are responsible for most foodborne illnesses [5]. The pathogenicity of these strains almost related to the ability of the microorganism to produce the one or two cytotoxins called Shiga toxins (*stx1* and *stx2*), and the presence of the intimin (*eae*) gene, which is responsible for intimate attachment of the organism to the intestinal epithelium [5,6].

Previous studies have demonstrated that antibiotic resistance levels in STEC are increasing. In addition, there is a great probability of association between the presence of virulence factors and antibiotic resistance [5,7]. When, at the same time, the presence of virulence factors with antibiotic resistance in bacteria happens, the antibiotics use may potentially increase the selection of bacteria carrying virulence genes, accelerating the expansion of virulence genes within bacteria [8].

Assumpção *et al*. [5] reported from 561 *E. coli* isolates, 90 (16.0%) were carriers of *stx1*, 97 (17.3%) of *stx2*, and 45 (8.0%) of *eae* genes singly. 37 (6.6%) isolates were carriers of *stx1* and *stx2*, 110 (19.6%) were carriers of *stx1* and *eae*, and 67 (11.9%) were carriers of *stx2* and *eae*. Other studies showed multidrug resistance (MDR) *E. coli* in food animals, milk and dairy products, and meat and meat products [9-11].

Therefore, the aims of this study were to investigate the prevalence and molecular characterization of *E. coli* in traditional milk products collected from different localities and markets located in Kashan of Iran.

## Materials and Methods

### Ethical approval

No ethical approval was required as no live animals were used in this study.

### Sample collection

A total of 116 samples of various traditional milk products including ice cream (n=60), yoghurt (30), and cheese (n=26) were purchased from randomly selected retail markets located in Kashan, Iran, from March 2016 to August 2016. All samples were immediately transferred to the food microbiology laboratory, Kashan University of Medical Sciences, in portable insulated cold-boxes. The samples were analyzed on the day they were collected.

### Isolation and identification of *E. coli*

A 10 g portion of each food sample was added to 90 mL of nutrient broth (Merck Co., Darmstadt, Germany) and stomached for 1 min. From the homogenate, 0.1 mL aliquots were spread plated on the MacConkey and EMB agar (Merck Co., Darmstadt, Germany) in duplicate following incubation for 24±2 h at 35±0.5°C. The phenotypic confirmation of the isolates as *E. coli* was done using Gram’s staining, catalase, oxidase, triple sugar iron agar, and IMViC tests [12].

### Antimicrobial susceptibility

Antimicrobial susceptibility testing of the *E. coli* and *Salmonella* isolates was performed by the Kirby-Bauer disc diffusion method using Mueller-Hinton agar (Merck Co., Darmstadt, Germany) according to the Clinical and Laboratory Standards Institute (CLSI) [13]. The following used antibiotics were tested: Ampicillin (10 µg), amoxicillin/clavulanic acid (30 µg), gentamycin (10 µg), tetracycline (30 µg), trimethoprim/sulfamethoxazole (10 µg), ceftazidime (30 µg), ciprofloxacin (10 µg), nitrofurantoin (300 µ*g*), norfloxacin (30 µ*g*), kanamycin (30 µ*g*), ceftriaxone (30 µg), and chloramphenicol (30 µg) (HiMedia Laboratories Pvt. Ltd, Mumbai, India). Briefly, a bacterial suspension with equivalent turbidity to 0.5 McFarland standard (1.5 × 10^8^ CFU/mL) was prepared in sterile phosphate buffered saline (PBS) (137 mM NaCl, 10 mM phosphate, 2.7 mM KCl, pH 7.4). The sterile swab stick was dipped into bacterial suspension and then on the surface of agar was uniformly inoculated. Afterward, antibiotic disks were placed for each plate and incubated at 35°C for 24 h. Inhibition zones on agar plate were measured, and the results were recorded in accordance with interpretive criteria provided by CLSI.

### Molecular methods

For extraction DNA from *E. coli* isolates, 1 mL overnight brain heart infusion (Merck Co., Darmstadt, Germany) broth culture (approximately 10^8^ bacteria), was centrifuged at 4,000 rpm for 5 min. The pellets were then washed in 1 mL of PBS, resuspended in 0.5 mL of H_2_O, and boiled (100°C) for 15 min. The cell debris was pelleted by centrifugation at 12,000 rpm for 5 min, and the supernatant containing the released DNA was transferred into a fresh microtube. The DNA purity and concentration were measured by a NanoDrop-1000 (NanoDrop Technologies, Wilmington, DE, USA).

The presence of genes encoding for the 16S RNA, *stx1*, *stx2*, and *eae* were detected by multiplex polymerase chain reaction (PCR) using specific primers (Takapou Zist Company, Tehran, Iran) (Table-1) [6,14].

**Table-1:** Primers used for polymerase chain reaction identification of *E. coli*, *stx1*, *stx2,* and *eae* genes.

Target gene	Primer	Sequence	Base pair	Reference
*E. coli*	Eco 2083 F	5′- GCTTGACACTGAACATTGAG-3′	662	[14]
	Eco 2745 R	5′- GCACTTATCTCTTCCGCATT-3′			
*stx1*	*stx1*-F	5′- CAACACTGGATGATCTCAG-3′	349	[6]
	*stx1*-R	5′- CCCCCTCAACTGCTAATA-3′	
*stx2*	*stx2*-F	5′- ATCAGTCGTCACTCACTGGT-3′	110	[6]
	*stx2*-R	5′- CTGCTGCTGTCACAGTGACAA-3′		
*eae*	*eae*-F	5′- GTGGCGAATACTGGCGAGACT-3′	890	[6]
	*eae*-R	5′- CCCCATTCTTTTTCACCGTCG-3′		

E.coli=Escherichia coli

The multiplex PCR procedure was performed according to Hessain *et al*. [6]. The PCR reaction mix included 2 μL of DNA, 5 μL of 10× PCR buffer, 2 μM dNTPs, 20 μM of each primer, and 2U Taq DNA polymerase and the volume of this mix was adjusted to 50 μl with sterile water. For multiplex PCR, the amplification was carried out in a thermal cycler (Eppendorf Master Cycler^®^, MA) with the following thermal cycling profile: An initial denaturation at 95°C for 5 min was followed by 30 cycles of amplification (denaturation at 95°C for 30 s, annealing at 50°C for 40 s, and extension at 72°C for 30 s), ending with a final extension at 72°C for 5 min. All PCR amplification products were separated on 1.5% agarose gel and visualized by staining with ethidium bromide using an ultraviolet light transilluminator.

### Statistical analysis

Chi-square and Fisher’s exact tests were used for the analysis of associations using SPSS software version 14. A p<0.05 was considered to be statistically significant.

## Results

From 116 samples collected of traditional milk products, it was isolated 11 (9.48%) *E. coli* strains. It was found that the incidence of *E. coli* in 60 ice creams, 30 yoghurt, and 26 cheese samples was 8.33%, 10%, and 11.54%, respectively (Table-2).

**Table-2:** Characterization of the recovered *E.coli* by multiplex PCR from traditional dairy product samples.

Types and number of collected traditional dairy product samples	*E.coli* (%)	Multiplex PCR of *E. coli*				

		*stx1*	*stx2* (%)	*eae*	*stx1* and *stx2* (%)	*stx1,* and *stx2 eae*
Ice cream (60)	5 (8.33)	5	5	0	5	0
Yoghurt (30)	3 (10)	3	3	0	3	0
Cheese (26)	3 (11.54)	3	3	0	3	0
Total (116)	11 (9.48)	11 (9.48)	11 (9.48)	0	11 (9.48)	0

*E.coli=Escherichia coli,* PCR=Polymerase chain reaction

**Figure-1:**
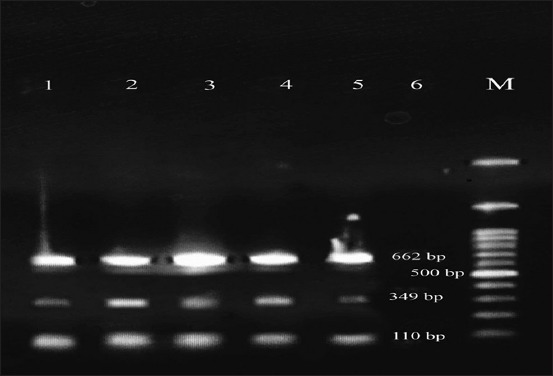
Gel picture is showing multiplex polymerase chain reaction-amplified products. M, 100-bp DNA marker; lane 1-6, 16S RNA *Escherichia coli*, *stx1*, and *stx2* positive; lane 6, negative control.

All isolates identified by cultural method were confirmed as *E. coli* by PCR (Figure-1). Furthermore, the results showed that 11 out of 11 (100%) *E. coli* had both *stx1* and *stx2* while *eae* gene was not found in *E. coli* isolated of traditional milk products (Table-2).

The frequencies of resistance to antibiotics were verified with all *E. coli* strains carrying *stx1* and *stx2* as virulence factors. For *E. coli* strains carrying *stx1* and *stx2*, highest antibiotic sensitive levels were related to trimethoprim/sulfamethoxazole, norfloxacin, chloramphenicol, and ciprofloxacin, respectively (Table-3). The 1 (9.1%), 4 (36.36%), 4 (36.36%), and 2 (18.18%) *E. coli* strains were resistant to 9, 10, 11, and 12 antibiotics, respectively.

**Table-3:** Frequency of resistance to antimicrobial agents among *E. coli* isolates harboring *stx1* and *stx2* from traditional dairy product samples (n=116).

Antimicrobial agent	Ice cream (%)	Yoghurt (%)	Cheese (%)	Total (%)
Ampicillin	5 (100)	3 (100)	3 (100)	11 (100)
Amoxicillin – clavulanic acid	5 (100)	3 (100)	3 (100)	11 (100)
Ceftazidime	5 (100)	3 (100)	3 (100)	11 (100)
Ceftriaxone	5 (100)	3 (100)	3 (100)	11 (100)
Kanamycin	5 (100)	3 (100)	3 (100)	11 (100)
Gentamicin	5 (100)	3 (100)	3 (100)	11 (100)
Tetracycline	5 (100)	3 (100)	3 (100)	11 (100)
Trimethoprim/sulfamethoxazole	0 (0)	2 (66.6)	1 (33.3)	3 (27.27)
*Norfloxacin*	2 (40)	2 (66.6)	1 (33.3)	5 (45.45)
*Nitrofurantoin*	5 (100)	3 (100)	3 (100)	11 (100)
*Chloramphenicol*	5 (100)	2 (66.6)	3 (100)	10 (90.9)
Ciprofloxacin	4 (80)	3 (100)	3 (100)	10 (90.9)

E. coli=Escherichia coli

The results showed that a statistically significant association was detected between antibiotic resistance and virulence genes in this *E coli* population isolated of traditional milk products (Table-4).

**Table-4:** Pairwise statistical associations between antimicrobial agents and virulence genes.

Antimicrobial agent	*stx1*(%)	*stx2*(%)	*eae*	p value*
Ampicillin	11 (100)	11 (100)		p=0.00
Amoxicillin – clavulanic acid	11 (100)	11 (100)		p=0.00
Ceftazidime	11 (100)	11 (100)		p=0.00
Ceftriaxone	11 (100)	11 (100)		p=0.00
Kanamycin	11 (100)	11 (100)		p=0.00
Gentamicin	11 (100)	11 (100)		p=0.00
Tetracycline	11 (100)	11 (100)		p=0.00
Trimethoprim/sulfamethoxazole	3 (27.27)	3 (27.27)		p>0.05
Norfloxacin	5 (45.45)	5 (45.45)		p>0.05
Nitrofurantoin	11 (100)	11 (100)		p=0.00
Chloramphenicol	10 (90.9)	10 (90.9)		p=0.02
Ciprofloxacin	10 (90.9)	10 (90.9)		p=0.02

-=Not detected *eae* gene in *E. coli* isolates. *E. coli=Escherichia coli*

The eleven *E. coli* isolates (100 %) were resistant to one or more antibiotics. All of the *E. coli* isolates were resistant to amoxicillin/clavulanic acid, ampicillin, nitrofurantoin, tetracycline, kanamycin, and gentamycin. Only eight of *E. coli* isolates were sensitive to trimethoprim/sulfamethoxazole, and six isolates were sensitive to norfloxacin (Table-3).

## Discussion

Hence, many of people are consuming unpasteurized milk and traditional products made from it. Increased nutritional qualities, flavor, and benefits have all been supported as reasons for interest in raw milk consumption [3]. Nevertheless, Claeys *et al*. [15] reported the occurrence of a number of pathogenic bacteria in raw milk and their products, including *Bacillus cereus*, *Brucella* spp., *Campylobacter* spp., pathogenic *E. coli*, *Listeria monocytogenes*. Therefore, consumption of raw milk and traditional milk products can be caused foodborne pathogens outbreaks.

In this work, 116 random samples of traditional milk products were investigated for the presence of *E. coli*. It is evident from the results demonstrated in Table-2 that the prevalence of *E. coli* in traditional milk products was 9.48%. Furthermore, in our work, all of the strains were harbored both *stx1* and *stx2*. Interestingly, no strains were harbored *eae* gene.

The occurrence of *E. coli* in raw milk, Karish cheese, and Ras cheese in Egypt was 76.4%, 74.5%, and 21.7%, respectively. Out of 222 *E. coli* strains, 104 (46.8%) isolated carried one or more virulence genes. The prevalence of *stx1*, *stx2*, and *eae* genes was 0.9%, 0.9%, and 0.45%, respectively [16].

Our results are in agreement with those of Elhadidy and Mohammed [1], who reported that STEC incidence in 124 cheese samples was 11.29%. All recovered isolates harbored *stx2* gene 100% either single or in association with the *stx1* gene, while *stx1* was present in 64.28% of isolates. They reported that intimin gene (*eae*) was present in 21% of isolates, while in our study any isolates had not *eae* gene. The percentage of positive raw milk and milk products with STEC in other studies was 17.2% [17] and 12.5% [18]. Rey *et al*. [18] showed that 3 STEC carried *stx1* gene, 5 possessed *stx2* gene, and 1 both *stx1* and *stx2*. Whereas only O157:H7 serotype showed *eae* virulence gene.

The pathogenicity of STEC is attributed to the production of *stx1* and *stx2* as verocytotoxin [6]. A previous study reported that *stx2* is the most important virulence factor and most of hemolytic-uremic syndrome cases in humans are caused by STEC strains harboring *stx2* gene [1]. The *eae* gene is an accessory virulence factor for STEC that is thought to enhance the virulence of STEC, while some STEC strains not harboring *eae* gene have been shown to cause human illnesses [19,20]. Further, Douellou *et al*. [21] demonstrated that the virulence gene profiles of dairy products and human STEC strains were similar.

Our data revealed an association between virulence factors and antimicrobial resistance in *E. coli* strains isolated from traditional milk products. All of the MDR STEC harbored *Stx1* and *Stx2*. Nagachinta and Chen [7] reported an association between virulence factors and antimicrobial resistance in *E. coli* strains isolated from dairy cows.

Cergole-Novella *et al*. [22] showed that the highest resistance rates of STEC were identified for tetracycline (100%), streptomycin (78%), and trimethoprim-sulfamethoxazole (56%). Furthermore, all strains were harbored *stx* genes.

Assumpção *et al*. [5] demonstrated that the STEC isolates that carried *stx1* only showed highest antimicrobial resistance to streptomycin (85.6%), followed by kanamycin (72.2%), and nalidixic acid (70.0%). Furthermore, STEC strains carrying *stx2* only showed highest antimicrobial resistance to streptomycin (73.2%), followed by kanamycin and nalidixic acid (70.1%). However, no statistically significant association was detected between any antimicrobial resistance phenotype and virulence genes. In our study, the STEC isolates that carried *stx1* and *stx2*, the highest antimicrobial resistance levels were to ampicillin, amoxicillin/clavulanic acid, gentamycin, tetracycline, nitrofurantoin, and kanamycin (100%).

The presence of antimicrobial resistance with virulence genes among STEC strains is worrisome. Therefore, when relationship between virulence genes and antimicrobial resistance occurs the antimicrobial resistance might be selected through the antimicrobial use, and as a result, the virulence genes would be selected as well [3,23].

## Conclusion

Findings of this work suggest that various traditional milk products at retail markets located in Kashan harbor multidrug-resistant *E. coli* which underscores the need of implementation of hygienic practices during production, transportation, and storage. Moreover, further researches are needed to study the incidence and prevalence of other harmful microorganisms and impact of their toxins produced in this product.

## Authors’ Contributions

RSC designed the study. FSC, NMA, and AN collected and processed the samples for isolation and identification of bacteria. RSC, EA, and AN were done PCR and electrophoresis. FSC and RSC interpreted the results and analyzed the data. RSC prepared the manuscript. All authors read and approved the final manuscript.
